# Mst1 regulates post-infarction cardiac injury through the JNK-Drp1-mitochondrial fission pathway

**DOI:** 10.1186/s11658-018-0085-1

**Published:** 2018-05-08

**Authors:** Xisong Wang, Qing Song

**Affiliations:** Department of Critical Care Medicine, the Chinese PLA General Hospital, Beijing, China

**Keywords:** Cardiac post-infarction injury, Mst1, Mitochondrial fission, JNK-Drp1 pathway, Cardiomyocyte death

## Abstract

**Background:**

Post-infarction cardiac injury is closely associated with cardiac remodeling and heart dysfunction. Mammalian STE20-like kinase 1 (Mst1), a regulator of cellular apoptosis, is involved in cardiac remodeling in post-infarction heart, but the mechanisms remain poorly defined. We aimed to explore the role of Mst1 in regulating chronic post-infarction cardiac injury, with a focus on mitochondrial homoeostasis.

**Methods:**

Wild-type (WT) and Mst1-knockout mice were as the cardiac myocardial infarction model. Cardiac fibrosis, myocardial inflammation response, heart dysfunction and cardiomyocyte death were measured in vivo using immunohistochemistry, immunofluorescence, western blot, qPCR and TUNEL assays. Cardiomyocytes were isolated from WT and Mst1-knockout mice, and a chronic hypoxia model was used to induce damage. Mitochondrial function was determined via JC1 staining, ROS measurement, cyt-c leakage detection and mitochondrial apoptotic pathways analysis. Mitochondrial fission was observed using immunofluorescence. A pathway activator and inhibitor were applied to establish the signaling pathways involved in regulating mitochondrial homeostasis.

**Results:**

Our study demonstrated that Mst1 expression was significantly upregulated in the heart post-infarction. Activated Mst1 induced cardiac fibrosis, an excessive inflammatory response, and cardiomyocyte death, whereas the genetic ablation of Mst1 protected the myocardium against chronic post-infarction injury. Function assays showed that upregulation of Mst1 activity contributed to JNK pathway activation, which led to Drp1 migration from the cytoplasm onto the surface of the mitochondria, indicative of mitochondrial fission activation. Excessive mitochondrial fission caused mitochondrial fragmentation, resulting in mitochondrial potential collapse, ROS overproduction, mitochondrial pro-apoptotic leakage into the cytoplasm, and the initiation of caspase-9-mediated mitochondrial apoptosis. By contrast, Mst1 deletion helped to maintain mitochondrial structure and function, sending pro-survival signals to the cardiomyocytes.

**Conclusions:**

Our results identify Mst1 as a malefactor in the development of post-infarction cardiac injury and that it acts through the JNK-Drp1-mitochondrial fission pathway.

## Background

Myocardial infarction (MI) is the leading cause of non-cancer mortality worldwide [1]. Timely reperfusion approaches, including coronary artery bypass grafting (CABG) and percutaneous coronary intervention (PCI), are standard post-MI treatments, but the limited ability of cardiomyocytes to regenerate damaged myocardial tissue results in the development of cardiac dysfunction [2].

Mechanistically, post-infarction heart remodeling and/or chronic cardiac damage result from cardiomyocyte death and subsequent cardiac fibrosis [3, 4]. First, MI causes excessive cardiomyocyte death via apoptosis or necrosis, leading to a decline in the number of functional cells over a short period [5, 6]. MI also initiates the chronic inflammatory response that increases the rate of cardiomyocyte death [7, 8]. In addition, MI activates cardiac fibroblasts, which produce excessive collagen and promote extracellular matrix accumulation [9].

The decreased number of functional cardiomyocytes and the increased amount of non-contractile tissue components collectively contribute to the progression of cardiac decompensation [10]. Therefore, a means to reduce cardiomyocyte death and alleviate cardiac fibrosis is essential to retard or delay the onset of post-infarction cardiac injury.

Mitochondria are the energy center of cardiomyocytes [11]. An increasing number of studies have identified mitochondrial damage as the pathogenesis for cardiomyocyte death and heart dysfunction. If the cardiomyocytes are deprived of energy due to mitochondrial injury, well-coordinated contraction cannot be guaranteed. Mitochondrial damage is accompanied by cellular oxidative stress or calcium overload, which lead to cardiomyocyte dysfunction [12–14]. Extensive mitochondrial stress is closely associated with cardiomyocyte apoptosis due to the liberation of pro-apoptotic factors (such as cyt-c) from the mitochondria into the cytoplasm [15]. For example, cyt-c interacts with and activates the apoptotic executor, caspase-3 [16, 17].

With its regulatory role in cardiac dysfunction and cell death, mitochondrial damage is considered the primary target for controlling the development of post-infarction cardiac injury. Interestingly, recent studies have found that mitochondrial damage in cardiomyocytes is mainly triggered by mitochondrial fission in response to MI [15]. Due to the coronary obstruction and nutrition shortage, mitochondria divide into several sister mitochondria to meet the energy requirement [15, 18]. However, excessive mitochondrial fission causes an uneven distribution of mitochondrial DNA in the sister mitochondria, most of which cannot then produce ATP but aggravate the cellular damage via multiple mechanisms [19]. This shows that inhibiting mitochondrial fission is necessary to sustain mitochondrial homeostasis and promote cardiomyocyte survival in the context of post-infarction cardiac injury.

Mammalian STE20-like kinase 1, a key component of the Hippo pathways [20], has received considerable attention as a regulator of acute and chronic cardiac injuries, such as ischemia reperfusion (IR) injury, diabetic cardiomyopathy and myocardial hypertrophy [21–23]. Convincing experimental data show the harmful effect of Mst1 on cardiovascular disease. Mst1 has also been reported to promote cardiac fibrosis and cardiomyocyte death in cases of post-infarction cardiac injury [22]. However, the mechanism underlying these effects remains unclear. More importantly, there is an incomplete understanding of whether Mst1 acts via regulation of mitochondrial homeostasis in post-infarction cardiac injury. The aim of our study is to explore the role of Mst1 in repairing the infarcted heart, with a focus on mitochondrial fission.

## Methods

### Myocardial infarction model

Wild-type (WT) and Mst1-knockout mice (Mst1^KO^) mice with a C57BL/6 background were purchased from K&D Gene Technology (WuHan) based on information from a previous study [22]. The mice were 12 weeks old and were housed under standard laboratory conditions (27 °C, 40–60% humidity, a 12-h light and dark cycle) with fresh drinking water and a commercial pellet diet. The MI mouse model was created by passing a 7–0 silk suture underneath the left anterior descending coronary artery with a knot, as described in a previous study [4]. After 28 days, the hearts were isolated.

### Chronic hypoxia model in vitro

Chronic hypoxia was applied to cardiomyocytes in vitro to mimic chronic post-infarction cardiac injury. This treatment was previously reported to be effective in imitating post-infarction injury in vitro [3]. Cardiomyocytes were isolated from the WT and Mst1^KO^ mice with trypsin and collagenase as described previously [11]. The cardiomyocytes were placed into a hypoxic incubator (95% N_2_ and 5% CO_2_) at 37 °C for approximately 48 h.

### Sample preparation and histological analysis

The hearts were excised and rapidly frozen in Optimal Cutting Temperature medium at room temperature (Agar Scientific Ltd.) for the preparation of 4-μm thick frozen sections. Masson trichrome staining was performed at room temperature and the sections were observed with an inverted microscope (magnification, 40×; BX51; Olympus Corp.) [24]. The levels of lactate dehydrogenase (LDH), troponin T, creatine kinase-MB (CK-MB), laminin and precollagen III in the blood were measured with ELISA assays as previously described [25].

### Echocardiography

Cardiac function was evaluated with an echocardiograph as previously described [26]. The mice were anesthetized using 3% isoflurane inhalation and studied using a Sequoia Acuson 15-MHz linear transducer echocardiography system (Siemens). The left ventricular ejection fraction (LVEF), left ventricular fraction shortening (LVFS), E/A ratio and left ventricular volume in systole (LV vol-s) were calculated using computer algorithms.

### Electron microscopy

Tissues were fixed at 4 °C with 2% glutaraldehyde in 0.1 mol/l sodium cacodylate buffer and post-fixed for 1 h on ice with 1% osmium tetroxide. The 60-nm sections were rinsed with distilled water and dehydrated using acetonitrile and graded methanol (50%, 20 min; 70%, 20 min; 95%, 20 min; and 100%, 20 min), and then embedded in epoxy resin (EMbed-812; Electron Microscopy Sciences) and polymerized at 70 °C overnight [27]. The sections were 60 nm thick and stained with lead citrate and uranyl acetate. The samples were imaged using a Hitachi H600 Electron Microscope. At least 30 cells in 5 randomly selected fields were observed [28].

### Immunofluorescence staining

The samples were first washed with cold PBS and then permeabilized in 0.1% Triton X-100 for 10 min at 4 °C. Then, 10% goat serum albumin (Invitrogen) was used to block the samples for 1 h at room temperature. The samples were incubated with primary antibodies overnight at 4 °C [29]. After three rinses in PBS, secondary antibodies were added to the samples for 1 h at room temperature [30]. The primary antibodies were: mitochondrial import receptor subunit TOM20 homolog (Abcam; cat. no. ab78547), F4/80 (1:1000, Abcam, #ab111101), troponin T (1:1000, Abcam, #ab8295), ICAM1 (1:1000, Abcam, #ab119871) and cyt-c (1:1000, Abcam, #ab ab133504). Images were observed with an inverted microscope (magnification, 40×; BX51; Olympus Corp.).

### Western blotting

Total protein (40–60 μg) was loaded onto a 12–15% SDS-PAGE gel. After electrophoresis, the proteins were transferred to a PVDF membrane (Roche Applied Science) [31]. Bands were detected using an enhanced chemiluminescence substrate (Applygen Technologies, Inc.). Band intensities were normalized to the respective internal standard signal intensity (β-actin, 1:2000; Abcam; cat. no. ab8224) [25]. The primary antibodies were: Mst1 (1:1000, Cell Signaling Technology, #3682), Bax (1:1000; Abcam; #ab32503), Bcl2 (1:1000, Cell Signaling Technology, #3498), Bad (1:1000; Abcam; #ab32455), caspase-9 (1:1000, Cell Signaling Technology, #9504), survivin (1:1000, Cell Signaling Technology, #2808), Mst1 (1:1000, Abcam, #ab184154), JNK (1:1000; Cell Signaling Technology, #4672), p-JNK (1:1000; Cell Signaling Technology, #9251), pro-caspase-3 (1:1000; Cell Signaling Technology, #9662), cleaved caspase-3 (1:1000; Cell Signaling Technology, #9664), Drp1 (1:1000, Abcam, #ab56788), TGFβ (1:1000, Abcam, #ab92486), MMP9 (1:1000; Cell Signaling Technology, #3852).

### RNA isolation and qPCR

Trizol reagent (Invitrogen) was used to isolate total RNA. The Eurogentec Reverse Transcription Kit was applied to transcribe RNA (one μg in each group) into cDNA. Quantitative PCR was performed with primers and matched probes from the Roche Universal Fluorescence-labeled Probe Library. The primers were: TNFα (forward, 5’-AGATGGAGCAACCTAAGGTC-3′; reverse, 5’-GCAGACCTCGCTGTTCTAGC-3′), IL6 (forward, 5’-CAGACTCGCGCCTCTAAGGAGT-3′; reverse, 5’-GATAGCCGATCCGTCGAA-3′), MCP1 (forward, 5’-GGATGGATTGCACAGCCATT-3′; reverse, 5’-GCGCCGACTCAGAGGTGT-3′) [2].

### Cellular ROS

To observe the cellular ROS levels, the ROS probe (DHE, Molecular Probes) was incubated with the cells for 30 min at 37 °C in the dark [32]. The cells were then washed with PBS to remove the ROS probe and immediately analyzed under a fluorescence microscope [33].

### mPTP opening assay, JC-1 staining and ATP production

mPTP opening is an early event in mitochondrial apoptosis. In our study, mPTP opening was measured via tetramethylrhodamine ethyl ester fluorescence. Samples were washed with PBS three times and then were loaded with tetramethylrhodamine ethyl ester (TEE). The fluorescence of TEE was recorded at the start and after 30 min. As detailed in a previous study [34], the mPTP opening rate was determined based on the time taken for the fluorescence intensity to decrease to half of the baseline.

Mitochondrial potential was assessed using a JC-1 probe, which is a sensitive fluorescent dye used to detect alterations in mitochondrial potential [11]. Following treatment, cells were incubated with 10 mg/ml JC-1 for 10 min at 37 °C in the dark and monitored with a fluorescence microscope (magnification, 100×; BX51; Olympus Corp.) [33]. Red-orange fluorescence was attributable to potential-dependent dye aggregation in the mitochondria. Green fluorescence, reflecting the monomeric form of JC-1, appeared in the cytosol following mitochondrial membrane depolarization [35].

ATP production was detected to reflect mitochondrial function. The samples were washed with cold PBS three times. Then the samples were lysed and a luciferase-based ATP assay kit (Beyotime Institute of Biotechnology) was used. ATP production was measured using a microplate reader [13].

### LDH assay and caspase-3 and -9 activity detection

LDH is released into the medium when cellular membranes rupture. To evaluate the LDH level in the medium, an LDH Release Detection kit (Beyotime Institute of Biotechnology) was used.

To analyze changes in caspase-3 and caspase-9, caspase-3/− 9 activity kits (Beyotime Institute of Biotechnology) were used according to the manufacturer’s protocols [36]. To analyze caspase-3 activity, 5 μl of DEVD-p-NA substrate (4 mM, 200 μM final concentration) was added to the samples for 2 h at 37 °C. To measure caspase-9 activity, 5 μl of LEHD-p-NA substrate (4 mM, 200 μM final concentration) was added to the samples for 1 h at 37 °C. The wavelength at 400 nm was recorded using a microplate reader to reflect the caspase-3 and caspase-9 activities [37].

### MTT and TUNEL assays

MTT experiments were performed in 96-well plates. Samples were washed 3 times with PBS and 50 μl of MTT reagent was added to each well. The samples were subsequently incubated for 4 h at 37 °C in a humid atmosphere containing 5% CO_2_. The MTT solution was removed, 200 μl of dimethyl sulfoxide was added to each sample, and the samples were incubated for 10 min [38, 39]. Following the addition of Sorensen’s buffer, the absorbance at the wavelength of 570 nm was determined.

To detect DNA fragmentation in the cell nuclei (a marker of apoptosis in testicular tissue), a TUNEL assay was performed using an In Situ Cell Death Detection kit (Roche Diagnostics GmbH) according to the manufacturer’s protocol. DAPI was used to label the nuclei (at room temperature for approximately 30 min) [40].

### Statistical analysis

All data are expressed as the means ± standard deviation. Statistical analyses were performed with SPSS software (version 17.0; SPSS, Inc., Chicago, IL, USA). The results from more than two groups were evaluated via one-way analysis of variance with the least significant difference test. *p* < 0.05 was considered statistically significant.

## Results

### Loss of Mst1 in post-infarcted hearts reduces cardiac fibrosis

**Fig. 1:**
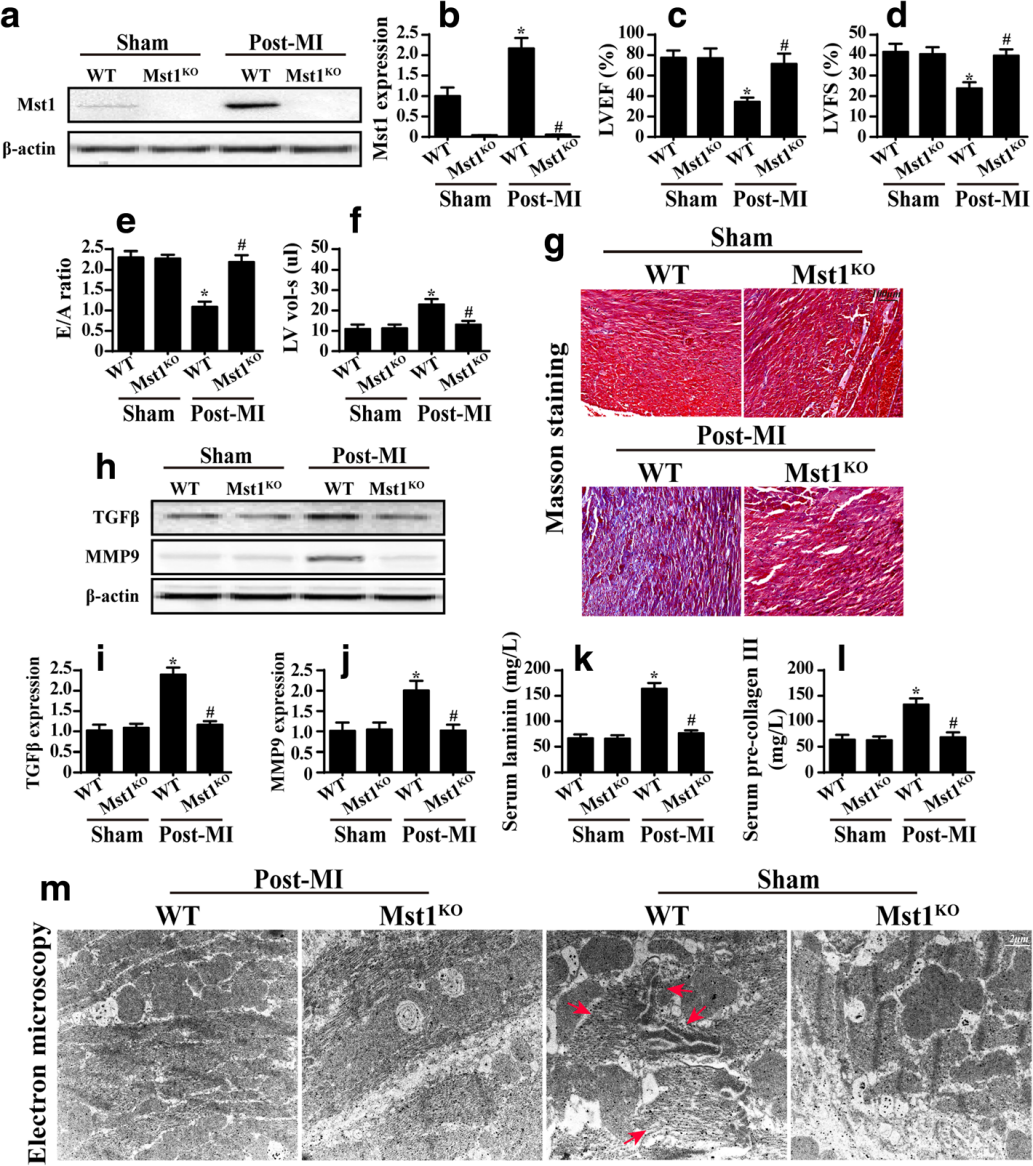
Mst1 levels were higher in the myocardium after a myocardial infarction (MI) and this contributed to the chronic cardiac damage. **a** and **b** The expression of Mst1 in the post-MI myocardium (Post-MI) in wild-type (WT) and Mst1-knockout (Mst1^KO^) cells relative to the control (Sham). **c**–**f** The cardiac function was detected as LVEF, LVFS, E/A ratio and LV vol-s via echocardiography in WT and Mst1^KO^ cells. **g** Masson staining was used to observe cardiac fibrosis. **h**–**j** The signaling pathways related to cardiac fibrosis (TGFβ and MMP9) were assessed via western blotting in WT and Mst1^KO^ cells. **k** and **l** The serum laminin and precollagen III levels in WT and Mst1^KO^ cells were measured via ELISA. **m** Electron microscope observations of the ultra-structural alterations in WT and Mst1^KO^ mice after MI. Red arrows indicated Z-line disappearance, cardiac muscle dissolution and cardiomyocyte disorganization. *p < 0.05 vs. sham group, ^#^*p* < 0.05 vs. WT mice in post-MI group

Western blotting was used to observe the change in Mst1 expression after myocardial infarction (MI). As shown in Fig. 1a and b, Mst1 expression was significantly upregulated 28 days after MI when compared to the results for the sham group myocardium.

We then asked whether an increased Mst1 level plays a causal role in cardiac dysfunction after MI. Mst1^KO^ mice were used for this set of experiments. Cardiac function was analyzed via echocardiography. We demonstrated that cardiac contractile function (LVEF and LVFS) was better in Mst1^KO^ mice than in the WT mice (Fig. 1c and d). Moreover, cardiac diastolic function (E/A ratio and LV volume) was also better in the Mst1^KO^ mice than in the WT mice (Fig. 1e and f). This information shows that Mst1 was activated by myocardial infarction and contributed to cardiac dysfunction.

Cardiac fibrosis is a key feature of infarcted hearts. Interestingly, Mst1 knockout reduced the level of cardiac fibrosis, as determined via Masson staining (Fig. 1g), showing that Mst1 is the cause of cardiac fibrosis. TGFβ and MMP9 expressions were higher in the heart after MI, but these phenotypic alterations were prevented by Mst1 knockout/were alleviated when Mst1 levels were reduced (Fig. 1h–j). The serum laminin and precollagen III concentrations (Fig. 1k–l) were notably increased in the samples from post-MI mice. Mst1 knockout reduced fibrosis marker levels.

To provide more solid evidence for Mst1-mediated cardiac damage, electron microscopy (EM) was used to observe the ultrastructural changes in the myocardium. The post-infarcted hearts showed Z-line disappearance, cardiac muscle dissolution and cardiomyocyte disorganization (Fig. 1m). These conformational alterations were recused by Mst1 deletion.

### Ablation of Mst1 expression alleviates cardiac inflammatory injury

**Fig. 2:**
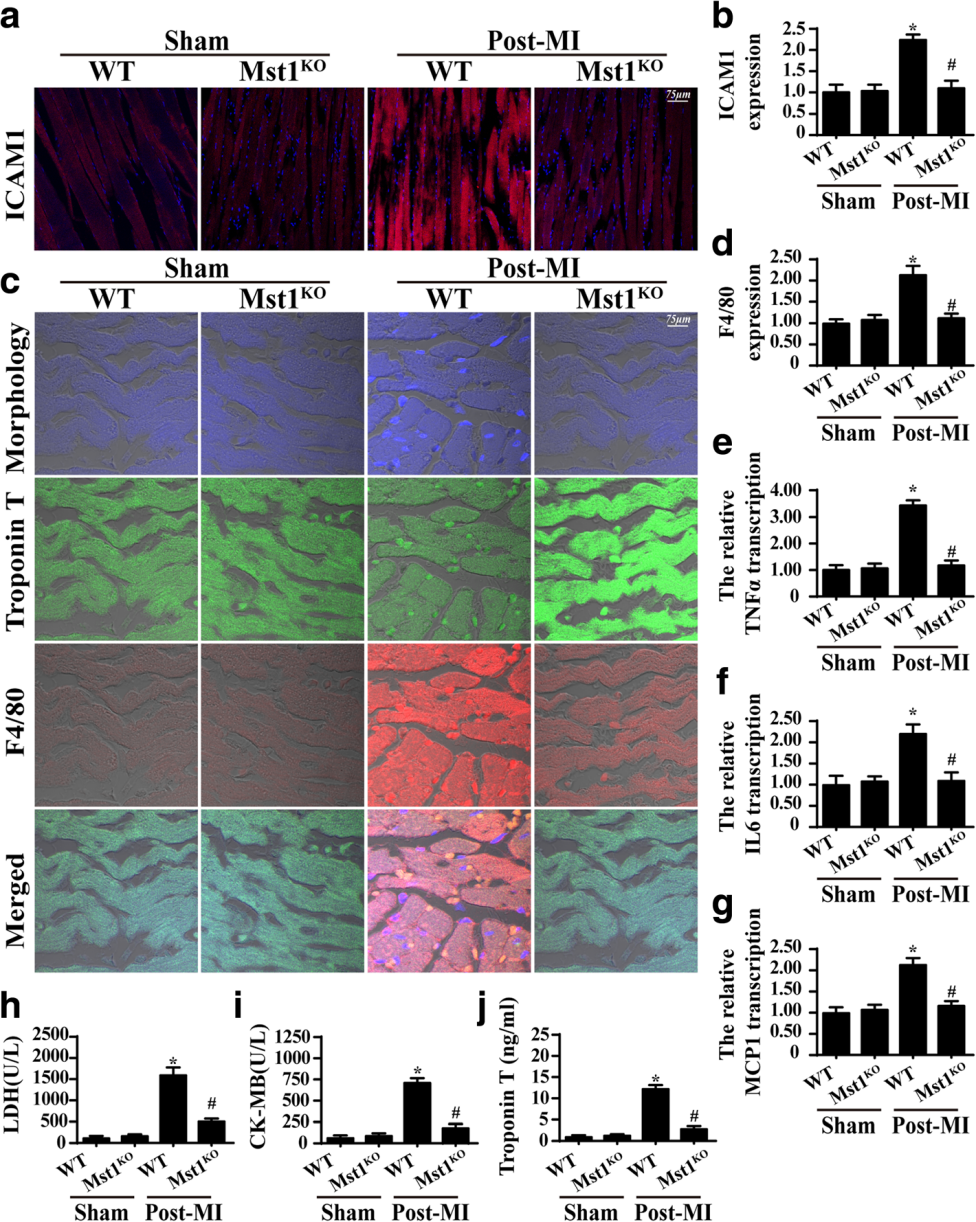
Mst1 regulated the inflammation response in the myocardium after a myocardial infarction (MI). **a** and **b** The expression of ICAM1 in myocardial tissue. **c** and **d** F4/80-positive macrophage migration into myocardial tissue was observed in wild-type (WT) cells post-MI using immunofluorescence assay. **e**–**g** The transcriptional alterations of inflammation factors (TNFα, IL6 and MCP1) in the myocardium post-MI. **h**–**j** The cardiac damage markers LDH, CK-MB and troponin-T were assessed in WT and Mst1-knockout (Mst1^KO^) cells via ELISA. *p < 0.05 vs. sham group, ^#^p < 0.05 vs. WT mice in post-MI group

The cardiac inflammatory response is also involved in the development of cardiac dysfunction in post-MI hearts. We demonstrated that the post-infarcted myocardium expressed more ICAM1 (Fig. 2a and b), which a kind of adherence factor that could capture the inflammation cells. By contrast, deletion of Mst1 reduced ICAM1 expression.

Through immunofluorescence assays of F4/80 macrophages, we demonstrated that more macrophages permeated the myocardial tissue of mice with post-MI hearts (Fig. 2c and d), indicating an excessive inflammatory response in the damaged heart. Mst1 knockout repressed the macrophage infiltration. As a result of cardiac inflammation, the IL6, TNFα and MCP1 transcription levels were also higher in post-infarcted myocardium (Fig. 2e–g), and this effect was inhibited by Mst1 knockout. This information indicates that Mst1 dysregulation is involved in the increased inflammatory response in post-MI hearts.

Excessive inflammatory injury induces cardiomyocyte injury, so we evaluated cardiac damage markers. Lactate dehydrogenase (LDH), troponin T and creatine kinase-MB (CK-MB) levels were significantly higher in the post-MI hearts than in the sham group hearts (Fig. 2h–j). However, Mst1 knockout reduced LDH, troponin T and CK-MB levels. These data indicate that Mst1 deletion is associated with a reduced inflammatory injury in post-MI hearts.

### Loss of Mst1 inhibits cardiomyocyte death

**Fig. 3:**
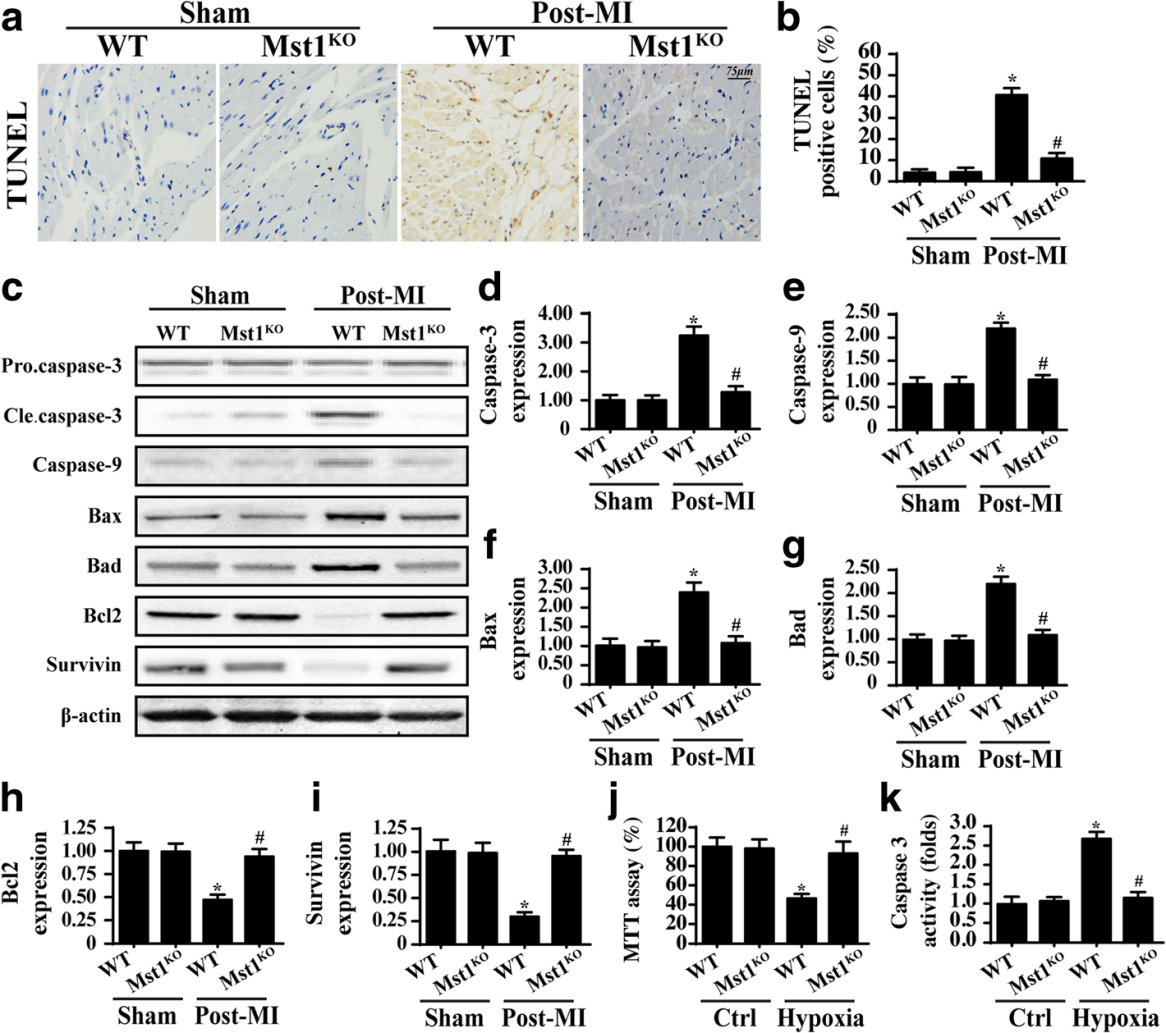
Mst1 promoted cardiomyocyte death in the heart after a myocardial infarction (MI). **a** and **b** The TUNEL assay was used to observe cellular apoptosis in vivo in wild-type (WT) and Mst1-knockout (Mst1^KO^) cells. **c**–**i** The mitochondrial apoptotic proteins pro-caspase-3, cleaved caspase-3 (Cle.caspase-3), caspase-9, Bax, Bad, Bcl2, survivin and β-actin were measured via western blots. Mst1 elevated mitochondrial apoptotic protein levels in the post-infarcted heart. **j** and **k** Cardiomyocytes were isolated from WT and Mst1^KO^ mice and a hypoxia model was used to induce cardiomyocyte damage in vitro. The MTT and caspase-3 activity assays were used to assess cellular viability *p < 0.05 vs. sham group, ^#^*p* < 0.05 vs. WT mice in post-MI group

Cardiomyocyte death is the primary risk factor in the progression of post-infarction cardiac injury. Through TUNEL assays, we found that post-MI hearts contained an higher number of TUNEL-positive cells (Fig. 3a and b). However, Mst1 knockout reduced the cardiomyocyte death ratio. Caspase-3, caspase-9, Bad and Bax expression levels were also higher in the post-MI heart (Fig. 3c–g). The levels of anti-apoptotic proteins such as Bcl2 and Survivin were lower (Fig. 3h and i). Interestingly, Mst1 knockout upregulated the level of anti-apoptotic factors and downregulated the expression of pro-apoptotic proteins in the post-MI heart (Fig. 3c–i).

To provide more solid evidence for the anti-apoptotic role of Mst1 deletion, cardiomyocytes were isolated from WT (WT cells) and Mst1^KO^ mice (Mst1^KO^ cells) and subjected to chronic hypoxia stress in vitro. Cell viability was detected via the MTT and caspase-3 activity assays. Compared to the control group, the chronic hypoxia group presented with reduced cell viability (Fig. 3j and k). This effect was reversed by Mst1 knockout. These data indicate that Mst1 deficiency attenuates cardiomyocyte death in the post-MI heart.

### Mst1 deletion sustains mitochondrial homeostasis

Mitochondrial damage is tightly linked to the cardiomyocyte death that occurs, at least in part, through cellular ROS release, mitochondrial potential collapse, mPTP opening and mitochondrial pro-apoptotic factor leakage.

**Fig. 4:**
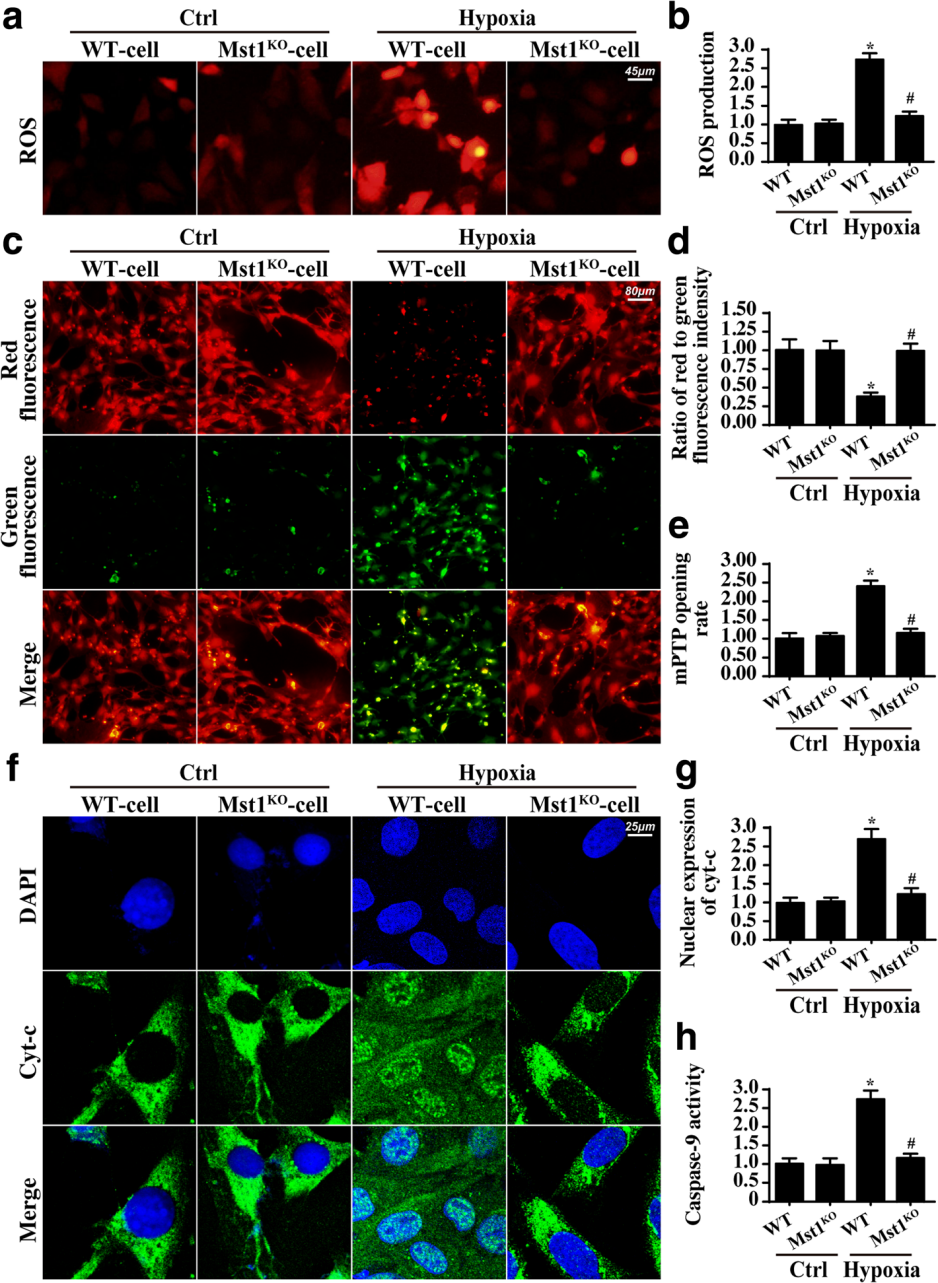
Mst1 impacted mitochondrial function and structure. **a** and **b** ROS production was higher in response to hypoxia treatment in wild-type cells (WT-cell) but was lower with Mst1 knockout (Mst1^KO^-cell). **c** and **d** The mitochondrial potential was measured via JC-1 staining. **e** The mPTP opening rate was evaluated and found to be highest in WT cells under conditions of hypoxia. Mst1 knockout meant mPTP opening rates closer to the normal level. **f** and **g** The immunofluorescence assay of cyt-c translocation from the cytoplasm (green staining) into the nucleus (blue staining). **h** Caspase-9 activity was measured to reflect mitochondrial apoptosis. *p < 0.05 vs. ctrl group, ^#^*p* < 0.05 vs. WT-cell+hypoxia

Through cellular ROS staining, we confirmed that chronic hypoxia stimulation increased ROS production in WT cells but not in Mst1^KO^ cells (Fig. 4a and b). The released ROS attack the mitochondrial membrane, leading to a reduction in mitochondrial potential.

Through JC-1 staining, we demonstrated that WT cells exhibited a dissipated mitochondrial potential in response to chronic hypoxia condition, as evidenced by a higher green fluorescence and lower red fluorescence (Fig. 4c and d). By contrast, Mst1^KO^ cells sustained their mitochondrial potential under chronic hypoxia conditions.

We also observed an increase in the mPTP opening rate in hypoxia-treated cells compared to that for the control group, and this tendency was reversed in Mst1^KO^ cells (Fig. 4e). Notably, mPTP opening provides a channel to facilitate the leakage of mitochondrial pro-apoptotic factors, such as cyt-c, into the cytoplasm. Through immunofluorescence assays, we illustrated that cyt-c diffusion into the nucleus increased after the hypoxia treatment, and that Mst1 knockout limited cyt-c migration (Fig. 4f and g). As a consequence, cytoplasmic cyt-c interacted with caspase-9 and increased caspase-9 activity, leading to caspase family activation and mitochondrial apoptosis. Through the analysis of caspase-9 activity, we uncovered that hypoxia stimulation enhanced caspase-9 activity (Fig. 4h). This effect was nullified by Mst1 knockout. Our data reveal that Mst1 deficiency protected cardiomyocytes against chronic hypoxia injury by maintaining mitochondrial homeostasis.

### Mst1 knockout abates excessive Drp1-mediated mitochondrial fission

Recent studies have reported that mitochondrial fission occurs during the early stage of mitochondrial apoptosis. Based on this, we asked whether mitochondrial fission is involved in cardiomyocyte mitochondrial apoptosis under chronic hypoxic stress.

**Fig. 5:**
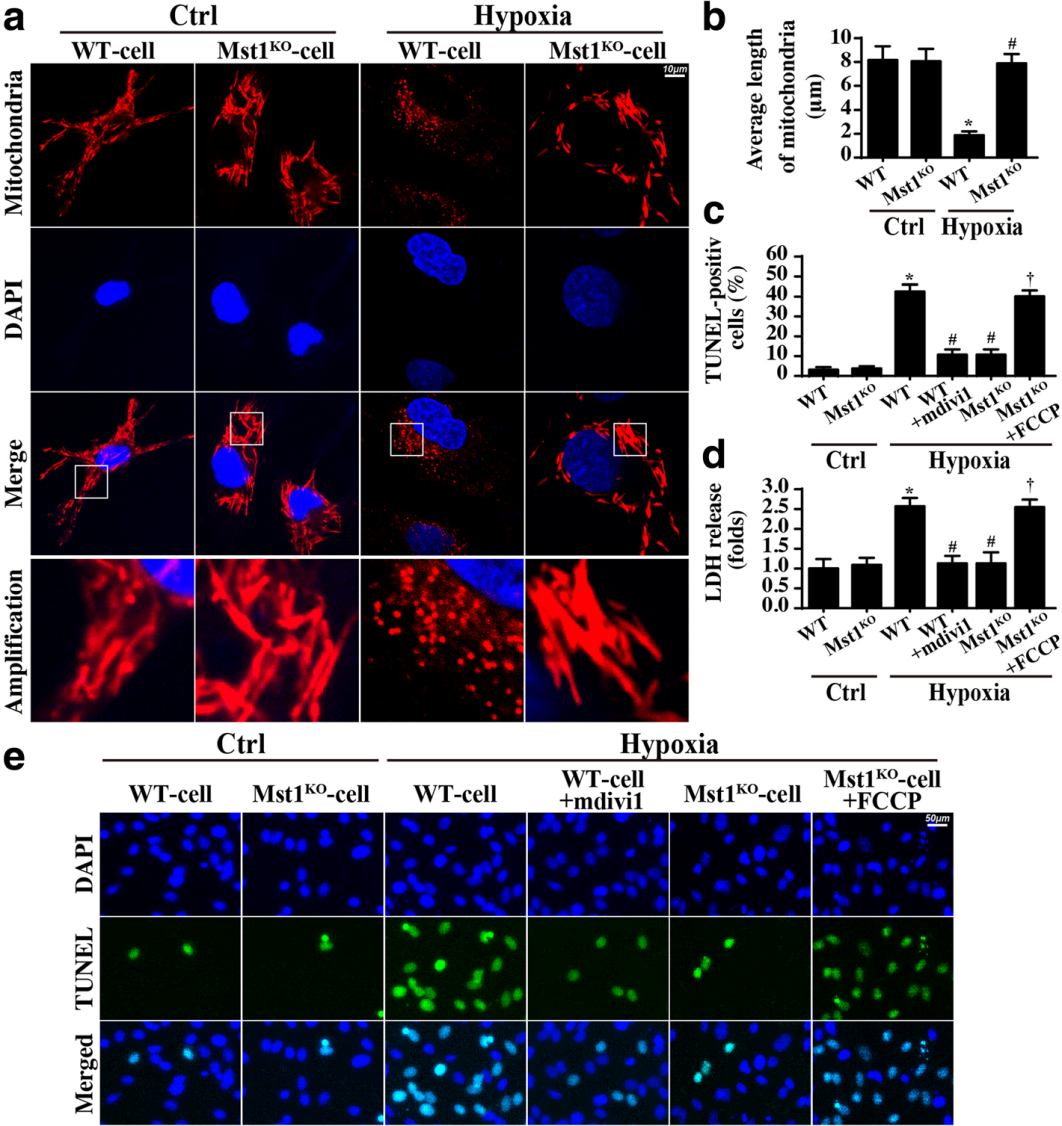
Mst1 controlled cellular apoptosis via mitochondrial fission. **a** The mitochondria of wild-type cells (WT-cell) and Mst1-knockout cells (Mst1^KO^-cell) were stained with Tom20 under control (Ctrl) and hypoxia conditions and mitochondrial fission was measured. **b** The average length of mitochondria was quantified. **c** and **e** The TUNEL assay was used to label the apoptotic cells. **d** The LDH release assay was carried out to measure the cardiomyocyte damage with mitochondrial fission activation and inhibition. *p < 0.05 vs. ctrl group, ^#^*p* < 0.05 vs. WT-cell+hypoxia, ^†^p < 0.05 vs. Mst1^KO^-cell+hypoxia

First, mitochondrial morphology was observed via confocal microscopy. As shown in Fig. 5a, compared to the control cardiomyocytes, the hypoxia-treated cardiomyocytes contained more punctate mitochondria that were smaller (significantly shorter long axes). However, most of the mitochondria in the Mst1^KO^ cells exhibited a long filamentous morphology, indicating the probable ability that Mst1 deletion suppresses hypoxia-induced mitochondrial fission. The average mitochondrial length was recorded to quantify the mitochondrial fission (Fig. 5b) and found to be 8.2 ± 1.3 μm in the control group, 1.9 ± 0.6 μm in WT cells after hypoxia treatment, and 7.8 ± 0.9 μm in Mst1^KO^ cells after hypoxia treatment. This suggests that mitochondrial fission was activated by hypoxia via Mst1 in cardiomyocytes.

To investigate whether fission is responsible for cardiomyocyte death, LDH release and TUNEL assays were performed. In hypoxia-treated cells, Mdivi1, a fission inhibitor, was used to inhibit hypoxia-activated fission. By comparison, FCCP, a fission activator, was administered to Mst1^KO^ cells to promote mitochondrial fission. Interestingly, blocking fission reduced LDH release and the number of TUNEL-positive cells, which is similar to the results obtained with Mst1 knockout (Fig. 5c–e). However, fission activation re-increased the LDH content and the ratio of TUNEL-positive cells despite Mst1 ablation (Fig. 5c–e). These data indicated that Mst1-mediated mitochondrial fission contributes to cardiomyocyte death under chronic hypoxic stress.

### Mst1 governs mitochondrial fission via the JNK-Drp1 pathway

**Fig. 6:**
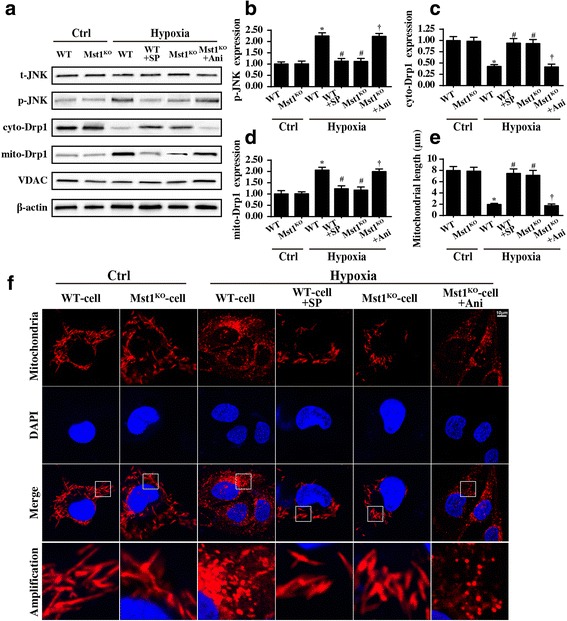
Mst1 regulated mitochondrial fission via the JNK-Drp1 pathway. **a**–**d** Western blotting was performed to analyze the signaling pathways. SP600125 (SP) was used to inhibit JNK activation (WT + SP). Anisomycin (Ani), an activator of JNK, was applied in Mst1-knockout cells to reactivate the JNK (Mst1^KO^ + Ani). **e** and **f** Mitochondrial fission and mitochondrial length were measured with JNK activation and inhibition. *p < 0.05 vs. ctrl group, ^#^*p* < 0.05 vs. WT-cell+hypoxia, ^†^*p* < 0.05 vs. Mst1^KO^-cell+hypoxia

Mitochondrial fission is finely regulated by Drp1, which migrates to and locates on the surface of mitochondria. It can form a ring-structure around the mitochondria and divide the mitochondria into several fragments via GPT-dependent contraction. We monitored the subcellular Drp1 localization via western blotting. Compared to the control group, hypoxic stress induced more Drp1 migration from the cytoplasm to the mitochondria (Fig. 6a–d), i.e., we observed an increase in mito-Drp1 and a decrease in cyto-Drp1. Mst1 knockout restored the cyto- and mito-Drp1 balance.

Previous studies have suggested that the Drp1 migration to the surface of mitochondria is regulated via JNK pathways. Interestingly, hypoxic stress activated JNK, as evidenced by an increase in phosphorylated JNK expression (Fig. 6a–d). This conformational alteration was inhibited by Mst1 deletion. To illustrate whether JNK activation accounts for Mst1-mediated Drp1 mitochondrial translocation, a JNK activator and inhibitor were used.

In hypoxia-treated cells, SP600125 (SP), an inhibitor of JNK, strongly alleviated JNK phosphorylation, reducing mito-Drp1 expression, which is similar to the results for the Mst1^KO^ cells (Fig. 6a–d). By contrast, anisomycin-mediated JNK reactivation in Mst1^KO^ cells via increased JNK phosphorylation/activation, and this was accompanied by increased mito-Drp1 expression (Fig. 6a–d). This illustrates that Drp1 mitochondrial localization is regulated via the JNK pathway in cardiomyocytes in the context of chronic cardiac damage.

To provide more direct support for the regulatory role of JNK in mitochondrial fission, mitochondrial morphology was observed again. JNK inhibition prevented mitochondrial fragmentations and maintained “normal” mitochondrial length, which is similar to the results for Mst1-deleted cells (Fig. 6e and f). By contrast, anisomycin-mediated JNK reactivation enhanced mitochondrial fission despite the Mst1 deficiency (Fig. 6e and f). Our data confirmed that excessive mitochondrial fission is controlled via the Mst1-JNK-Drp1 signaling pathway.

## Discussion

A growing body of evidence suggests the involvement of the Mst1-Hippo pathway in cancer proliferation, cellular migration, cardiac ischemia reperfusion and diabetic cardiomyopathy [41–43]. However, little is known about the role of Mst1 in chronic cardiac injury after myocardial infarction (MI).

In this study, we confirmed that:

1. Mst1 is significantly upregulated in the myocardium after MI
2. Mst1 knockout alleviated cardiac fibrosis, the excessive inflammatory response and cardiomyocyte death
3. At the molecular level, Mst1 knockout favored cardiomyocyte survival and sustained mitochondrial homeostasis by inhibiting mitochondrial fission
4. Mst1 knockout reduced JNK activation and Drp1 mitochondrial translocation, effectively inhibiting fatal mitochondrial fission

To the best of our knowledge, this is the first paper to describe the role of Mst1 in post-infarction cardiac injury and to show the involvement of the JNK-Drp1-mitochondrial fission pathway.

Mst1, a downstream factor of the Hippo pathway, has been implicated in acute and chronic cardiovascular disorders. Excessive Mst1 activation aggravates acute ischemia reperfusion injury by augmenting cardiomyocyte oxidative stress [44], promotes cardiac hypertrophy by enhancing cardiomyocyte necrosis [45], and induces diabetic cardiomyopathy [21] by inhibiting protective autophagy. In addition, Mst1 is associated with the survival, development and metastasis of colorectal cancer [20], non-small cell lung cancer [46] and hepatocellular carcinoma [47].

Our study also found that Mst1 expression was much higher in the myocardium post-MI and that this contributed to post-infarction cardiac injury through the promotion of cardiomyocyte mitochondrial apoptosis. These data validate Mst1 as a vital regulator of cellular survival, highlighting that Mst1 may be a target for sustaining cardiomyocyte viability in response to acute and/or chronic stress damage [48, 49].

We found that excessive mitochondrial fission was involved in cardiomyocyte death in the post-MI heart. Moderate mitochondrial fission into sister mitochondria is activated to meet the increased metabolic requirements of cardiomyocytes [50–52]. However, based on our data, excessive mitochondrial fission induced mitochondrial dysfunction, as evidenced by uncontrolled oxidative stress, reduced mitochondrial potential, increased mPTP opening and extensive cyt-c leakage. This concurs with the findings of an earlier study that identified mitochondrial fission as the pathogenesis for cardiac acute ischemia reperfusion [53, 54].

Notably, earlier studies [6, 15] argued that mitochondrial fission caused mitochondrial DNA damage, cardiolipin oxidation and hexokinase 2 liberation, finally activating the mitochondria-dependent apoptotic pathway. Interestingly, another study suggested that excessive mitochondrial fission impaired mitophagy, ultimately resulting in the accumulation of damaged mitochondria [5, 55].

Based on this information, we concluded that mitochondrial fission lies upstream of mitochondrial damage, including mitophagy, mitochondrial DNA integrity and mitochondrial apoptosis. This makes mitochondrial fission an early hallmark of cardiomyocyte damage. However, more clinical evidence is needed to support our conclusion [56].

We also demonstrated that the JNK-Drp1 pathway is responsible for mitochondrial fission. Drp1 migration from the cytoplasm onto the mitochondrial surface is a prerequisite for successful mitochondrial fission [15, 57]. Several researchers have explored the molecular basis for Drp1 migration. The AMPK, JNK, DUSP1 and p53 pathways have been verified as the molecular machinery for Drp1 mitochondrial translocation [6, 16, 18, 19, 58]. In cardiac reperfusion and liver cancer, JNK activation is associated with excessive Drp1-mediated mitochondrial fission [59–61].

Our results provide the first evidence for the direct role of JNK in Drp1-mediated mitochondrial fission in post-infarction cardiac injury. Notably, whether JNK could indirectly interact with Drp1 and influence Drp1 activity remains unclear. Further research is needed.

## Conclusion

We explored the role and mechanism of Mst1 in post-infarcted myocardial injur. Upregulation of Mst1 activated the JNK-Drp1 mitochondrial fission pathway, sending mitochondrial apoptotic signals to the cardiomyocytes. Based on this conclusion, novel therapeutic approaches that regulate the balance between the Mst1 level and mitochondrial homeostasis may improve the prognosis of patients after a myocardial infarction.

## Abbreviations

Cyt-c: Cytochrome c
Drp1: Dynamin-related protein 1
MI: Myocardial infarction
mPTP: Mitochondrial permeability transition pore
Mst1: Mammalian STE20-like kinase 1
